# Role of Statin Drugs for Polycystic Ovary Syndrome

**Published:** 2016-12

**Authors:** Lisa Cassidy-Vu, Edwina Joe, Julienne K Kirk

**Affiliations:** 1 Department of Family and Community Medicine, Wake Forest School of Medicine, Winston-Salem, North Carolina, United States; 2 College of Pharmacy and Health Sciences, Campbell University, Buies, Creek; 3 Diabetes and Endocrinology Center, Department of Family and Community Medicine, Wake Forest School of Medicine, Winston-Salem, North Carolina, United States

**Keywords:** Polycystic Ovarian Syndrome, PCOS, Hydroxymethylglutaryl-CoA Reductase Inhibitors, Statin Drugs

## Abstract

**Objective:** To review the potential role and specific impact of statin drugs in women with PCOS. The evidence for this use of statins in PCOS is limited and still under further investigation.

**Materials and methods:** A search was conducted using PubMed, DynaMed and PubMedHealth databases through October 16, 2016 using the terms *polycystic ovary syndrome, PCOS, hydroxymethylglutaryl-CoA reductase inhibitors, hydroxymethylglutaryl-CoA, statin, atorvastatin, fluvastatin, lovastatin, pitavastatin, pravastatin, rosuvastatin and simvastatin*. English-language trials evaluating statins in PCOS were obtained and incorporated if they provided relevant data for providers.

**Results:** We summarize twelve trials involving statins in PCOS. The trials were predominantly 12 weeks to 3 months in length (8 of the 12 trials) and low to moderate dose of statin drugs were used. The majority (10 of 12) of the trials show that statins reduce testosterone levels or other androgen hormones (DHEA-S and androstenedione), half of the trials evaluating LH/FSH ratio show an improvement, and all had positive effects on lipid profiles.

**Conclusion:** Statins show promising improvements in serum levels of androgens and LH/FSH ratios translating to improved cardiovascular risk factors above and beyond simply lowering LDL levels. More investigation is needed to determine if statins can clinically impact women with PCOS long term, particularly those who are young and are not yet candidates for traditional preventative treatment with a statin medication.

## Introduction

Polycystic ovarian syndrome (PCOS) is widely accepted as the most common endocrine abnormality in women of childbearing age, with a prevalence in the United States of 6 to 15% depending on which criteria is used for diagnosis (1). There is not a universally accepted definition, but rather three expert groups have weighed in on the diagnosis of PCOS. The National Institute of Health criteria requires both hyperandrogenism (either clinically or biologically) and oligo- or amenorrhea (less than 10 menses in a year) (2). This definition is more rigid and captures a severe phenotype. Another expert group includes the Rotterdam Consensus which requires two of three criteria consisting of hyperandrogenism, oligo- or amenorrhea, or polycystic ovaries (3). The Androgen Excess Society and PCOS Society require hyperandrogenism and either oligo- or amenorrhea or polycystic ovaries (4). By these definitions, a broader number of women are classified as PCOS. The evidence to support metabolic complications from PCOS is evolving. 

There are multiple characteristics seen in women with PCOS that increase their risk of atherosclerotic cardiovascular disease (ASCVD) versus the general population. Calcification of coronary arteries in PCOS women is more prevalent and severe (4). The size of the left ventricle as well as the stiffness of the ventricle is increased (5). Women with PCOS have elevated homocysteine, a marker of inflammation and endothelial cell injury linked to atherogenesis (4).Higher levels of total cholesterol, low density lipoproteins (LDL), very low density lipoproteins (VLDL), and triglycerides are also associated with PCOS (6). A lower level of high density lipoproteins (HDL) is seen in PCOS women and is the most common lipid abnormality. Increased prevalence of obesity and hyperandrogenism in PCOS more readily leads to metabolic syndrome, increasing the risk of diabetes (7). Overall, there is a three to seven fold risk of myocardial infarction in women with PCOS (4, 5). Furthermore, other inflammatory markers such as cytokines and adipokines as well as C-reactive protein (CRP) levels have been linked to cardiac health. There are studies that show a relationship between elevated levels of these inflammatory markers and PCOS, which adds to the ASCVD risk of these women (4, 8). 

With regards to these cardiovascular risk factors, a practitioner may ask whether there is a preventative benefit to providing a statin to PCOS women of childbearing age, particularly in those less than 40 years, for which ASCVD risk score cannot be calculated. The other practice issue is how providers approach the risk of placing women of childbearing age on medication that may provide benefit but also carries a teratogenic risk at conception.

There are currently no formalized recommendations or guidelines for statin use in PCOS women without dyslipidemia, but American Congress of Obstetricians and Gynecologists (ACOG) recommends a statin for prevention of ASCVD (2). Statins are well known for reducing LDL. Other effects of statins include a reduction of inflammatory markers and improving both menstrual irregularities and clinical signs of androgen excess (9-12). We sought to specifically search the potential role and impact of statin drugs in PCOS.

## Materials and methods

**Figure 1:**
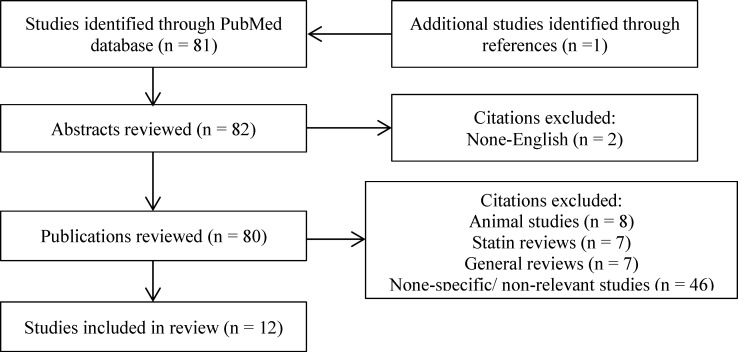
Flowchart of study inclusion

***Data Sources and Study Selection:*** A search of PubMed was conducted through October 16, 2016 to identify clinical studies pertaining to the use of statins in PCOS women. Search terms included polycystic ovary syndrome, PCOS, hydroxymethylglutaryl-CoA reductase inhibitors, hydroxymethylglutaryl-CoA, statin, atorvastatin, fluvastatin, lovastatin, pitavastatin, pravastatin, rosuvastatin, and simvastatin. Additional databases were searched during the process, including Dyna Med and PubMed Health. We looked at citations of clinical trials and review articles. Bibliographies of these studies and review articles were also examined. All English-language trials examining the use of statin drugs in PCOS were evaluated. Figure 1 outlines a flowchart of study inclusion for this review.

***Properties of Statins:*** Hydroxymethylglutaryl-CoA (HMG-CoA) reductase inhibitors, also known as statins, work by competitively inhibiting HMG-CoA reductase. As the rate-controlling enzyme of the mevalonate pathway, HMG-CoA reductase is essential in the pathway for the synthesis of cholesterol. By blocking this enzyme, the conversion of HMG-CoA to mevalonate is prevented and thus, the synthesis of cholesterol is blocked (11).

Statins are widely known for their lipid-lowering profile. These drugs lower LDL cholesterol by reducing hepatic cholesterol production and increasing LDL clearance from the blood. HDL cholesterol and triglyceride concentrations are also improved with the use of statins. While the statin class of drugs has been shown to be beneficial in the lipoprotein aspect, they also have associated anti-inflammatory properties (9, 10). Reduction of inflammatory markers (i.e. CRP), typically elevated in PCOS, has prompted investigation of the role of statin drugs as a treatment option.

Adverse effects of statins can include headache, difficulty sleeping, flushing of the skin, drowsiness, dizziness, nausea or vomiting, abdominal cramping or pain, bloating or gas, diarrhea, constipation, andrash (13). Mild myalgia may affect 5 to 10% of statin users while rhabdomyolysis and statin-induced necrotizing autoimmune myopathy are rare (14). Another infrequent adverse effect of statins is liver toxicity which is moreconcerning in the setting of active liver disease (11). Statin-associated symptoms also include a small relative and absolute risk of elevated blood glucose and type 2 diabetes (14). Reports of cognitive impairment from post-marketing data suggest that statins may cause drug-induced memory loss or dementia which is reversible upon drug discontinuation (14).

## Results

***Clinical Studies*** *: *Statin drugs have been studied in a variety of trials for PCOS (Table 1-3). Many of the studies have compared the use of a statin in patients using oral contraceptive pill (OCP) or with placebo and/or metformin (15- 26).More than one statin has also been evaluated in a head-to-head comparison (25). Twelve studies met our search criteria that report outcomes of statin therapy in PCOS (15- 26) that are reviewed. 

***Simvastatin:*** Several randomized trials have been conducted with simvastatin in women with PCOS (Table 1). Duleba and colleagues (15) investigated simvastatin with an OCP (20 ugethinyl estradiol and 150 ugdesogestrel) versus an OCP alone for 12 weeks in 48 women (24 in each group) with PCOS. This trial was followed by a crossover study with the same group of patients (16). Cholesterol profiles improved in the statin group while testosterone levels decreased more with simvastatin compared with OCP alone. Hirsutism was significantly improved with simvastatin and there was no significant decrease in subjects on OCP alone. The studies found that simvastatin did not affect adrenal steroidogenesis as evidenced by no effect on dehydroepiandrosterone sulfate (DHEAS). Simvastatin did impact the hypothalamo-pituitary axis.Between the groups, there was a difference in the response of gonadotropin levels where luteinizing hormone (LH) levels decreased more in the statin group while follicle stimulating hormone (FSH) did not significantly change. Markers of systemic inflammation including CRP and soluble vascular cell adhesion molecule (sVCAM) were decreased by simvastatin (16). Limitations in both of the studies by Duleba and colleagues (15,16)include the homogeneity of the groups of women (young, white, with lower body mass index (BMI) and initial insulin levels), short study duration, and no randomization to a statin-only arm.

A study by Banazsewska, et al included a trial using metformin, simvastatin, or the combination in 113 PCOS subjects for 3 months (17). As expected, cholesterol levels decreased more in the simvastatin groups compared to metformin alone (Table 1). A decline in testosterone and hirsutism was seen in all three groups but simvastatin alone was superior in its ability to lower compared to metformin or the combination of simvastatin/metformin. The limitations of this trial were the short duration (12 weeks) and the participants were of lower BMI and age. The same authors then extended the study for 6 months and demonstrated that simvastatin was superior to metformin and improved menstrual regularity along with a reduction of ovarian volume (18). Cholesterol profiles showed significant improvement of total cholesterol in the simvastatin treated group. In the two studies (17, 18), there was no placebo arm and the subjects along with the providers were not blinded.

Kazerooni and colleagues (19) conducted a randomized, placebo controlled study using simvastatin plus metformin versus metformin plus placebo among 84 women with PCOS over 12 week. A significant decrease in testosterone, LH, LH:FSH ratio, total cholesterol, triglyceride, LDL levels, and hirsutism were found in the simvastatin plus metformin group (Table 1). 

**Table 1:** Simvastatin Use in PCOS

**Author/ (Year)**	**N (average age +/- SD)/ Study Design/ Duration**	**Meds Used**	**Cholesterol Outcomes**	**Other Findings**	**Labs**
Duleba AJ, et al.^15^ (2006)	48 PCOS women (statin group: 24 +/- 0.7; OCP group: 23.8 +/- 0.8), prospective randomized trial, 12 week study	Simvastatin (Sim) + OCP vs. OCP alone	-TC decreased by 10% vs. an 8% increase -LDL decreased by 24% vs. a 3% increase -HDL increased by 9% vs. 13% -TG increased by 5% vs. 21%	-Serum T levels decreased by 41% vs. 14% (p=0.006) -LH/FSH decreased by 44% vs. 12% (p=.02) -LH level decreased by 43% vs. 9% (p=0.02) -Ferriman-Gallweyhirsutism score declined by 0.25+/- 0.1 points (p=0.03) vs. 0.13 +/- 0.09 (P=0.2)	Serum T, LH, FSH, and prolactin levels, sex hormone-binding globulin, DHEAS, TC, TG, LDL
Banaszewska B, et al.^16^ (2007) Crossover study of Duleba AJ, et al.^15^ (2006)	48 PCOS women (statin group: 24 +/- 0.7; OCP group: 23.8 +/- 0.8), prospective crossover randomized trial, 12 week study	Sim (+OCP) vs. OCP alone (evaluated a total of 24 weeks of both crossover arms)	-TC and LDL decreased by 7.5% and 20%, respectively in statin group -OCP alone increased TC by 5% and no effect on LDL -Increases of HDL comparable in both groups -TG’s remained unchanged after -Statin + OCP group but increased 20% after OCP alone	-Total T decreased by 38% vs. 26% (p=0.004) -Free T decreased by 58% vs. 35% (p=0.006) -Ferriman-Gallweyhirsutism score decreased by 8.1% vs. 4.7% (p=0.02) -LH decreased by 37% vs. 18% (p=0.002) -LH:FSH decreased by 40% vs. 25% (p=0.01) -High sensitivity-CRP decreased by 45% vs. 6% (p=0.006)	Total T, free T, TC, TG, HDL, LDL, hs-CRP, LH, FSH, LS:FSH, prolactin
Banaszewska B, et al.^17^ (2009)	113 PCOS women (Sim group: 26.1 +/- 0.6; Metformin (Met) group: 25.2 +/- 0.7; Sim +Met group: 24.7 +/- 0.6), prospective randomized trial, 3 month study	Sim, Met, or Sim + Met (Sim Met)	-LDL decreased by 32% (Sim) and 40% (Sim Met) vs. a 2.1% increase (Met) -TC decreased by 20.6% (Sim) and 24% (Sim Met) vs. a 3.4% increase (Met) -TG decreased by 6.3% (Sim) and 22.7% (Sim Met) vs. a 0.6% increase (Met)	-Total T decreased in all groups – 16.3% (Sim) (p<0.001), 13.6% (Met) (p<0.01) and 15.1% (Sim Met) (p<0.001) -Free T decreased in all groups – 13.5% (Sim) (p<0.001), 13.1% (Met) (p=0.02) and 11.1% (Sim Met) (p<0.01) -Ferriman-Gallweyhirsutism score decreased by 1.8% (Sim Met) (p=0.03) vs.1.7% (Sim) (p=0.06) and 3.7% (Met) (p=0.06) -Hs-CRP decreased in all groups -34% (Sim) (p<0.01), 52% (Met) (p<0.01) and 47% (Sim Met) (p=0.01) -Insulin decreased by 17.4% (Sim) (p<0.01) vs. 5.4% (Met) (p=0.38) and 13.1% (SimMet) (p=0.15) & insulin sensitivity increased by 14% (Sim) (p<0.01) vs. 6.3% (Met) (p=0.4)	Total T, fasting insulin, insulin sensitivityCRP, DHEAS, LH, FSH, TC, LDL, BMI
					
Banaszewska B, et al.^18^ (2011) Extension study from Banaszewska B, et al.^17^ (2009)	139 PCOS women (Sim group: 26.3 +/- 0.6; Met group: 26 +/- 0.6; Sim +Met group: 25.3 +/- 0.6), prospective randomized trial, 6 month study	Simvastatin (Sim), metformin (Met), or simvastatin + metformin (Sim Met)	-LDL decreased by 31.6% (Sim) and 31.9% (Sim Met) vs. a 2.4% increase (Met) -TC decreased by 18.9% (Sim and Sim Met) vs. a 2.8% increase (Met) -TG decreased by 15% (Sim Met) vs. a 17.5% increase (Met) and a non-significant decrease in Sim -TC and LDL declined in subjects receiving Sim during first 3 months of treatment, with no significant change during next 3 months	-Volume of ovaries decreased by 14.1% (Sim) (p<0.0001) and 7.3% (Sim Met) (p=0.04) vs 5.4% (Met) (p=0.06), with no change from 3 to 6 months in Met group -Number of spontaneous menses increased 71.3% (Sim) vs. 33.1% (Met) (p=0.03) and 73.3% (Sim Met) vs. 33.1% (Met) (p=0.02) -Total T decreased in all groups – 25.6% (Sim) (p<0.0001), 25.6% (Met) (p<0.0001) and 20.1% (Sim Met) (p<0.0001) -Free T decreased in all groups – 20.3% (Sim) (p<0.0001), 23.2% (Met) (p<0.0001) and 17.5% (Sim Met) (p=0.003) -Ferriman-Gallweyhirsutism score decreased in all groups – 12% (Sim) (p<0.0001), 8.9% (Met) (p<0.0001) and 11.7% (Sim Met) (p<0.0001) -Fasting insulin decreased only in Sim Met by 20.9% (p=0.02) vs. an increase of 11.1% (Met)	Insulin, total and free testosterone, LH, FSH, prolactin, SHBG, DHEAS, change in total serum cholesterol, TG, HDL, LDL, hs-CRP
Kazerooni T, et al.^19^ (2010)	84 PCOS women (Met + Sim group: 25.6 +/- 4.23; Met + placebo group: 24.9 +/- 5.81), prospective randomized double-blind placebo-controlled, 12 week study	Met (500mg 3x daily) + Sim vs. Met (500mg 3x daily) + placebo	-TC decreased by 29.5% vs 4.2% -HDL increased by14% vs. 1% decrease -LDL decreased by 18.5% vs. 1.5% -TG decreased by 32% vs. 5.3%	-Total T decreased by 25.5% vs. 16.8% (p<0.001) -LH decreased by 46% vs. 10% (p=0.001) -LH:FSH ratio decreased by 38% vs. increased by 4% (p=0.009) -Ferriman-Gallweyhirsutism score decreased by 13% vs. 7% (p=0.019)	Total T, serum LH, FSH, PRL, DHEAS, TC, fasting blood sugar, fasting insulin, TC, HDL, LDL, TG
Krysiak R, et al.^20^ (2014)	14 PCOS women (ezetimibe group: 37 +/- 4; Sim group: 38 +/- 3), comparison study, 90 day study	Ezetimibe 10mg daily vs. Sim 40mg daily	-TC decreased by 21% vs. 23% LDL decreased by 29% vs. 24% -Neither group showed significantly altered levels of TG and HDL	Total T decreased by 23% vs. 7% (p<0.01) Free T decreased by 32% vs.14% (p<0.01)	Total T, free T, DHEAS, androstenedione, LH/FSH ratio, SHBG, prolactin, serum FSH and LH, LDL, TC, TG

BMI, body mass index; CV, cardiovascular; DHEAS, dehydroepiandrosterone sulfates; FSH, follicle stimulating hormone; HDL, high density lipoprotein; hs-CRP, high sensitivity C-reactive protein; LDL, low density lipoprotein; LH, luteinizing hormone; M, mean; Met, Metformin; OCP, oral contraceptive; Sim, Simvastatin; T, testosterone; TC, total cholesterol; TG, triglyceride.

**Table 2:** Atorvastatin Use in PCOS

**Author/Year**	**N (average age +/- SD)/ Study design/ Duration**	**Meds Used**	**Cholesterol Outcomes**	**Other Findings**	**Labs**
Sathyapalan T, et al.^21^ (2009)	40 PCOS women (atorvastatin group: 26.6 +/- 1.2; placebo group: 28.8 +/- 1.8), randomized double-blind placebo-controlled, 12 week study	Atorvastatin vs. placebo	-TC decreased by 26% vs. 2% -LDL decreased by 36% vs. no change -TG decreased by 21% vs. increased by 18% -No detectable changes in HDL	-Total T decreased by 25% (p < 0.01) -Hs-CRP reduced by 25% (p = 0.05) -Insulin levels decreased by 21% (p < 0.01)	TC, LDL, TG, hs-CRP, T levels
Sathyapalan T, et al.^22^ (2012) Extension Study of Sathyapalan T, et al.^21^ (2009)	40 PCOS women (atorvastatin group: 26.6 +/- 1.2; placebo group: 28.8 +/- 1.8), randomized double-blind placebo-controlled, 3 month study	Atorvastatin vs. placebo followed by subsequent 12 weeks of metformin	Not studied	-DHEAS decreased by 15% (p = 0.02) -Androstenedione decreased by 17% (p = 0.03) -No significant changes in either androstenedione or -DHEAS concentrations in placebo group after 12 weeks of metformin	DHEA, androstenedione
Raja-Khan N, et al.^23^ (2011)	20 women with PCOS (atorvastatin group: 33.8 +/- 4.3; placebo: 29.4 +/- 5.8), double-blind randomized placebo-controlled trial, 6 week study	Atorvastatin 40 mg daily vs. placebo	-TC decreased by 39% vs. 5% -No significant Hchange in HDL -LDL decreased by 51% vs. 9% -TG decreased by 49% vs. increased by 5%	-Androstenedione decreased by 26% vs. increased by 7% (p < 0.001) -DHEAS decreased by 18.6% vs. increased by 2% (p = 0.02) -Hs-CRP decreased by 46% (p = 0.02) vs. 16.7% -Insulin AUC worsened by 27% (p = 0.03) vs. improved by 2% (p = 0.79)	TC, HDL, LDL, TG, hs-CRP, androstenedione, total T, DHEAS, glucose/insulin, FMD
Puurunen J, et al.^24^ (2013)	28 PCOS women (atorvastatin group: 40.5 +/- 5.9; placebo group: 38.5 +/- 4.8), randomized double-blind placebo-controlled trial, 6 month follow-up study	Atorvastatin 20 mg vs. placebo	-TC decreased by 31% vs. 2% -LDL decreased by 45% vs. no change -TG decreased by 25% vs. increased by 9% -No significant change in HDL	-Fasting insulin levels higher from baseline by 9% at 6 months vs. lower at 1.4% (p = 0.023) -Stimulated AUCs of insulin higher from baseline by 0.2% at 6 months vs. lower by 18% (p = 0.023) -Insulin sensitivity (Matsuda index) decreased by 21% vs. increased by 12% (p = 0.002) -DHEAS decreased 15% (p < 0.001) vs. 10% -Total T did not significantly decrease in atorvastatin group but did in placebo by 22% (p = 0.009) -CRP decreased 23.5% vs. increased by 33% (p < 0.006)	Androstenedione, T, DHEAS, estradiol, LH, FSH, SHBG, CRP, TC, LDL, HDL TG, glucose
Kaya C, et al.^25^ (2009)	52 PCOS women (23.4 +/6.2), prospective randomized trial, 12 week study	Atorvastatin 20 mg vs. simvastatin 20 mg daily	-TC decreased by 21% vs. 25% -LDL decreased by 26% vs. 24% -TG not significantly decreased -HDL increased by 21% vs. no significant increase	-Total T decreased 36.5% (p < 0.01) vs. 46% (p < 0.01) -Free T decreased 38.3% (p = 0.05) vs. 40.6% (p < 0.01) -Homocysteine levels decreased by 26% (p < 0.01) vs. 18% (p < 0.05) -Fasting insulin decreased by 40% (p < 0.01) vs. no significant change	Serum homocysteine, free and total T levels, FSH, LH, DHEAS, HDL, LDL, TG, total cholesterol

AUC, area under curve; DHEAS, dehydroepiandrosterone sulfates; hs-CRP, high sensitivity C-reactive protein; FSH, follicle stimulating hormone, FMD, flow mediated dilation; HDL, high density lipoprotein; LDL, low density lipoprotein; M, mean; LH, luteinizing hormone; TC, total cholesterol; TG, triglyceride; T, testosterone.

**Table 3:** Rosuvastatin Use in PCOS

**Author/Year**	**N (M +/- SD)/ Study design/ Duration**	**Meds Used**	**Cholesterol Outcomes**	**Other Findings**	**Labs**
Ghazeeri G, et al.^26^ (2015)	37 PCOS women (rosuvastatin group: 25.9 +/- 6.5; rosuvastatin + metformin group: 25.7 +/- 5.4), prospective randomized double-blind placebo controlled, 6 month study	Rosuvastatin 10mg/day x 3 mos. Then rosuvastatin + metformin (Rosuvatatin+Metformin) 850mg/BID vs. Rosuvstatin + placebo x 3 months	-TC increased by 14% in Rosuvastatin+Metformin vs. 6% in Rosuvastatin (p = 0.005) LDL increased by 21% in Rosuvastatin+Metformin group vs. 11% in Rosuvastatin (p = 0.007) -No significant changes in HDL or TG	-No significant differences between intervention and control groups were found for CRP, homocysteine, DHEAS, testosterone and insulin -Fasting blood glucose levels increased by 5% vs. 1% (p = 0.02)	Homocysteine levels, testosterone, CRP, DHEAS, FBS, insulin, TC, HDL, LDL, TG

CRP, C reactive protein); DHEAS, dehydroepiandrosterone sulfates; FBS, fasting blood sugar; M, mean; PCOS, Polycystic Ovary Syndrome; HDL, high density lipoprotein; LDL, low density lipoprotein; LH, luteinizing hormone; TC, total cholesterol; TG, triglyceride.

The authors note that the effects seen on hyperandrogenism, insulin resistance, and lipid profile cannot be attributed to any one drug alone. The effect of a synergism should be considered. Limitations of this study include the short duration of three months and the effects from simvastatin on the ovary cannot be distinguished from those on the hypothalamus and pituitary.

Krysiak et al. (20) conducted a study of simvastatin, with another non-statin cholesterol lowering agent, ezetimibe, among 14 women with PCOS for 90 days. Simvastatin was superior to ezetimibe in reducing testosterone levels. Significant decreases in androstenedione, DHEAS, LH/FSH ratio were noted with simvastatin use. This study was limited by its short duration of 12 weeks, small sample size, and the ezetimibe group of women was preselected by their history of intolerance to a statin.

***Atorvastatin:*** Sathyapalan et al. (21) studied the effects of atorvastatin in 40 women with PCOS and biochemical hyperandrogenemia over a 12 week period. A significant reduction in hs-CRP, lipid profile and insulin levels were found with atorvastatin compared to placebo (Table 2). The identical therapy was then studied among the same group with the addition of metformin added to the regimen (22). DHEAS and androstenedione levels were significantly lowered. Both of these studies were of short duration (12 weeks), limiting the clinical impact on signs of hyperandrogenism (21, 22).

Atorvastatin has been compared to placebo among women with PCOS at varying doses and durations. Raja-Khan et al. (23) evaluated a small group of 20 women. A 45% reduction in hs-CRP compared to a 15% decrease in placebo was found but did not reach statistical significance. This study was terminated at 6 weeks due to funding. Another small study of 28 women with PCOS received atorvastatin over a six month follow-up and found that statin therapy worsened insulin sensitivity (24). This study was limited by a non-statistically significant increase in age and BMI in the atorvastatin group as well as higher baseline testosterone levels.

A randomized trial comparing atorvastatin to simvastatin was conducted by Kaya et al. (25) among 52 women with PCOS over a 12 week period. Both statins were found to lower testosterone at similar rates along with homocysteine, fasting insulin, TC, LDL, and LH that were significantly reduced compared to baseline. Homocysteine reduction was higher in the atorvastatin versus the simvastatin. The dose of each statin was modest (20 mg daily) compared to other studies that used slightly higher doses.

***Rosuvastatin:*** Ghazeeri et al. (26) evaluated rosuvastatin in 37 women with PCOS in combination with metformin or placebo (Table 3). The results indicated that there were no significant differences between the intervention and control group for lipid profiles, CRP, homocysteine, DHEAS, testosterone fasting plasma glucose or insulin. In fact, at month 6 in the intervention group, LDL, TC, and FBS were increased. The authors did not ask about compliance with treatment, the study was of small sample size, and both groups received rosuvastatin (no control group with only placebo).

## Discussion

Statin drugs are widely accepted as agents used in the prevention of ASCVD in patients with comorbidities such as diabetes and hyperlipidemia with an LDL greater than 190 or when the ASCVD risk is 5% or greater (5, 27).There is a plethora of evidence that statins reduce LDL and improve overall cholesterol profile (27, 28).There is not a guideline for the use of statins in PCOS despite growing evidence that PCOS is a risk factor for cardiovascular disease (29). A meta-analysis of statin use in PCOS among four of the studies identified in this review reported a significant decrease in testosterone (30). Higher testosterone levels have been shown in PCOS women to correlate with more atherogenic lipid profiles (31). Levels of free testosterone and clinical hyperandrogenism have been linked to an increased risk of metabolic syndrome (another ASCVD risk factor) in PCOS women (32).

***Statin Considerations for Use in PCOS:*** In women with clinical signs of hirsutism, the current options include oral contraceptives (OCPs), spironolactone and anti-androgen medications such as finasteride. Spironolactone has mixed data on effectiveness without concomitant OCP use and finasteride is not recommended in adolescents because of potential bone loss (33). Several reviews regarding treatment strategies advocate for the use of statin drugs in PCOS (34-38). Statins can lower testosterone levels in the studies reported (Tables 1-3) which in turn lead to less clinical signs of hyperandrogenism, giving clinicians another possible agent to treat acne and hirsutism.

Statins were shown to improve LH:FSH ratio (15, 16, 19, 20).This was seen in a head to head study (20) of simvastatin versus ezetimibe, in combination of simvastatin with OCPs (15, 16) versus OCPs, and in combination of simvastatin with metformin versus placebo plus metformin (19). In these latter three studies, it is possible that it is the synergistic effect of simvastatin and OCPs as well as the effect of simvastatin with metformin that improves the LH/FSH ratio. The statin drugs also decrease ovarian size, as seen in the head to head trial of simvastatin monotherapy versus simvastatin plus metformin versus metformin alone where both groups with simvastatin showed a decrease in ovarian size (17). These factors can improve the chronic anovulatory cycles that accompany PCOS. First, by creating a more normal LH: FSH balance, the ovaries are encouraged to produce estrogen over testosterone. Second, by not over stimulating the ovaries with an abundance of LH, one follicle is allowed to mature and be released. This can positively impact fertility in the PCOS woman.

Most importantly, statins improve lipid profiles. Statins decrease inflammatory markers such as CRP (16, 17, 21, 23) and homocysteine (24). Statin use decrease testosterone levels in all of the studies with simvastatin and most of the studies with atorvastatin (Tables 1 and 2) possibly through inhibiting the same mevalonate pathway as described earlier, thereby decreasing the cholesterol needed for androgen production (16). The combination of lowering LDL and triglycerides, increasing HDL, and improving inflammatory markers and testosterone levels can provide ASCVD protection to women with PCOS. 

Two potential controversial aspects of using statins in the PCOS population are 1) the impact on glucose levels, and 2) the teratogenicity of statins. In the studies reviewed, there were conflicting effects on glucose and insulin resistance. Two simvastatin studies (17, 18) demonstrated an improvement in fasting insulin levels while one atorvastatin study (21) showed a reduction in serum insulin levels. Two atorvastatin studies (23, 24) however, showed worsening hyperinsulinemia and the rosuvastatin study (26) showed significant hyperglycemia in the statin group. Since PCOS is a risk factor for diabetes, adding an agent that can potentially worsen insulin sensitivity and glucose levels is a concern that warrants further investigation.

Statins are rated as a high risk category in pregnancy and the majority of women are diagnosed in their childbearing years. More investigation about pregnancy risk is needed. The provider and the patient must agree that the statin be taken concomitantly with a reliable birth control method with frequent follow-up. As more data becomes available, the potential role of statin medicines, even for a limited time frame, might be an option. Augmentation with atorvastatin pretreatment before metformin therapy has also been studied (39). The use of atorvastatin prior to initiating metformin was associated with positive effects of metformin including decreased glucose resistance and increased insulin sensitivity even after discontinuing the statin. This may be an option that warrants more investigation for women who desire pregnancy but want to avoid any potential risk to the fetus with statin use.

To reflect the potential risk that PCOS infers on women, more research can be done to assess PCOS as a cardiovascular risk factor to aid clinicians in discussing the full implications of PCOS. Discussing ASCVD risk is of particular importance in women who are young and less likely to be interested in preventing a disease that will not affect them for many years. Integrating PCOS into tools such as the ASCVD risk calculator will assist clinicians in determining the need for statins in their female patients and warrants further consideration. Additionally, creating a guideline for the use of statins in PCOS may help decrease patient resistance and improve adherence to this class of medication. Given the prevalence of PCOS in the population, a guideline can also serve to provide an organized approach especially in light of the uncertainty in practice of treating women of childbearing age with a statin.
